# 

**Published:** 1917-08-25

**Authors:** 


### CONTENTS.

| | PAGE |
|---|---|
| **A Hospital Dispute in Tasmania...** | 407 |
| **A Vital Question for Some Voluntary Hospitals** | 408 |
| **Hospital and Institutional News ...** | 409 |
| The Limits of Institutional Interference—The Health of the Salonica Army—The Troops at Aden—A School for Hospital Managers—Warnings of Air-Raids—The Extension of Newcastle Infirmary—One Hundred Thousand Interested Hospital Workers—A New Shell-shock Hospital—Hospital Legacies—Large Gifts to Blind Charities—The Training of the Blind—Lectures on Tuberculosis—Plague at Gravesend—A Hospital She-goat—The Soldier and the Cigarettes—Politicians and Cottage Hospitals—Ashton District Infirmary—King Edward VII.'s Hospital—Pembrokeshire and the Welsh Memorial—The Chaplain of Bracebridge Asylum—The Lincolnshire County Asylum Board Cemetery—A Maternity Home for Morley—Westmorland and Child Welfare—Infant Welfare Results—The Underpayment of Dentists' Mechanics—Consumption in London—The Ministry of Labour and the Asylum Workers' Union—Dispensing with Glycerine and Sugar—This Week's Drug Market—To Our Readers. | |
| **The Strain of War on the Nervous System...** | 415 |
| Efficiency in Politicians and Soldiers. | |
| **London as a Health Centre** ... ... ... | 417 |
| By a Social Worker. | |
| **From Across the Seas** ... ... ... ... | 419 |
| **Matrons in Council** ... ... ... ... ... | 420 |
| A Rejoinder to "An Old Shoe's" Thoughts. | |
| **The Matrons' and Sisters' Department** ... | 421 |
| Matron's in Council: IV. The Love and Sympathy of a Noble Woman. | |
| Round the Hospitals. | |
| Injustice to Night Nurses—Rights of the Rank and File—Each Nurse's Own Standard—Halcyon Days Ahead for Nurses—Strong Position of Nightingale Fund—A Brave and Noble Sister. | |
| **A Jungle Hospital** ... ... ... ... ... | 425 |
| The Travelling Dispensary Officer's Work.—I. | |
| **Parliament and the Profession** ... ... ... | 426 |
| **Addenbrooke's Hospital, Cambridge** ... ... | 427 |
| **Matrons' and Sisters' Appointments** ... ... | 428 |
| **Institutional Appliances** ... ... ... | 428 |
| **The Institutional Worker** ... | (Sup pl.) |

